# Leadership micro-behaviors and performance stability under competitive pressure: a stepped-wedge field study in professional football

**DOI:** 10.3389/fpsyg.2026.1833589

**Published:** 2026-05-14

**Authors:** René Paasch, Gunnar Mau

**Affiliations:** 1 Department of Psychology, DHGS Deutsche Hochschule für Gesundheit und Sport, Berlin, Germany; 2 Department of Marketing and Retailing, Magdeburg-Stendal University of Applied Sciences, Magdeburg, Germany

**Keywords:** leadership, coaching behavior, psychological safety, collective efficacy, performance stability, elite sport, football

## Abstract

Leadership in elite sport is often assumed to directly enhance performance outcomes, yet empirical evidence remains inconsistent, particularly under conditions of high competitive variability. The present study examined whether a brief, behavior-focused PERMA-based leadership intervention was associated with changes in psychological team resources and performance stability in competitive football teams. Using a stepped-wedge field design, 10 teams were observed across up to 24 competitive weeks, combining longitudinal team-level panel data with intensive daily diary assessments (1,567 player-day observations). Fixed-effects and event-study models were used to estimate within-team changes following intervention onset. Perceived leadership micro-behaviors increased following intervention onset, alongside higher levels of psychological safety and collective efficacy. At the performance level, no consistent changes in match points were observed, whereas performance instability decreased, reflecting lower week-to-week fluctuation across matches. Additional robustness analyses were consistent with the interpretation that observed patterns were more closely aligned with reduced performance variability than with systematic changes in average performance levels. Moderation analyses suggested that these associations were attenuated under conditions of elevated competitive pressure. Daily diary results provided converging evidence for changes in leadership perception and psychological safety, but not for load-dependent experiential states such as flow or sleep. These findings suggest that leadership micro-behaviors may be associated with context-sensitive patterns of team functioning, particularly in relation to performance stability rather than immediate competitive success. By distinguishing between performance stability and outcome performance, the present study contributes to a more differentiated understanding of leadership in elite sport and highlights the potential relevance of behavior-focused interventions in applied competitive settings.

## Introduction

In professional team sports, leadership is widely assumed to shape how teams regulate performance under sustained competitive pressure, uncertainty, and public scrutiny (Cotterill and Fransen, 2016; Fransen et al., 2020). Beyond tactical decisions and training design, coaches influence the psychological context in which collective performance unfolds, particularly in environments characterized by time pressure, public evaluation, and rapidly shifting situational demands such as elite football (Arnold et al., 2012; Fletcher and Sarkar, 2012). Despite broad agreement on the importance of leadership in sport, empirical evidence on how specific leadership behaviors operate in real competitive environments remains limited (Fransen et al., 2020). This aligns with research highlighting the role of athlete leadership and shared leadership structures in team functioning (Fransen et al., 2015b).

Recent research has increasingly emphasized context-sensitive leadership processes in high-performance sport, highlighting the role of relational leadership, shared team identity, and psychological safety for collective functioning under competitive pressure (Fransen et al., 2020; Haslam et al., 2020). However, much of this work relies on cross-sectional designs or focuses on global leadership perceptions rather than observable behavioral practices enacted during ongoing competition periods. As a result, it remains unclear whether concrete leadership behaviors during competitive periods are associated with changes in shared team processes and functional performance regulation over time.

Research in sport psychology has primarily examined leadership through broader stylistic frameworks such as transformational leadership, motivational climates, and autonomy-supportive coaching (Callow et al., 2009; Mageau and Vallerand, 2003; Ntoumanis et al., 2017). Leadership has also been linked to positive developmental experiences in sport settings (Vella et al., 2012). Classic sport leadership research has also emphasized behavioral dimensions of coaching through multidimensional leadership models (Chelladurai, 1990; Chelladurai and Saleh, 1980). While these approaches have substantially advanced understanding of athlete motivation, engagement, and well-being, they provide limited insight into how leadership behaviors unfold dynamically across competitive periods (Stebbings et al., 2011; Fransen et al., 2015a). Consequently, it remains unclear whether leadership-related changes extend beyond individual perceptions to influence shared team functioning and performance regulation over time.

Recent work has further highlighted context-sensitive and psychologically informed coaching processes in high-performance environments, emphasizing the role of autonomy-supportive and relational leadership behaviors in shaping resilience, coping, and adaptive functioning under competitive pressure (e.g., Li et al., 2025).

While conceptually related to these frameworks, the present study adopts a more behaviorally granular perspective. While conceptually related to these frameworks, the present study adopts a more behaviorally granular perspective. While conceptually related to established leadership frameworks such as transformational leadership and autonomy-supportive coaching, the present approach differs in its level of analysis. Traditional leadership frameworks typically capture global leadership styles or athletes’ general perceptions of coaching behavior across situations (Callow et al., 2009; Mageau and Vallerand, 2003; Ntoumanis et al., 2017). In contrast, the concept of leadership micro-behaviors focuses on concrete, observable, and situation-specific coaching actions enacted in everyday training and competition contexts. This behavioral perspective allows for a more fine-grained examination of how leadership is enacted in real time and how such actions may relate to short-term psychological processes and performance regulation under competitive conditions. Leadership micro-behaviors refer to concrete, situation-specific coaching actions embedded in everyday training and competition. Rather than capturing global leadership styles, this approach focuses on observable behavioral enactment, allowing for a more precise examination of how leadership unfolds in real-time performance contexts.

From a performance psychology perspective, leadership effects are unlikely to manifest primarily in immediate match outcomes. Instead, leadership may operate through proximal regulatory processes that support coordination, communication, and collective functioning under uncertainty (Marks et al., 2001). Constructs such as psychological safety and collective efficacy represent key team-level resources facilitating adaptive responses to competitive stressors (Edmondson and Lei, 2014; Newman et al., 2017; Frazier et al., 2017; O’Donovan et al., 2020). In highly variable environments, performance stability may therefore represent a more sensitive outcome indicator than isolated match results.

In the present study, performance stability is operationalized as week-to-week fluctuation in team-level match performance (points and goal difference), calculated as absolute changes across consecutive matches. Lower values indicate more consistent performance trajectories. This approach allows leadership-related associations to be examined in terms of functional regulation across repeated competitive situations.

To structure observable leadership behaviors, the PERMA framework (Seligman, 2011) is used as a pragmatic organizing taxonomy rather than a theory of leadership. Conceptual work has also emphasized the role of well-being in competitive sport contexts (Lundqvist, 2011). This approach aligns with established sport leadership perspectives emphasizing observable coaching behaviors and interpersonal interaction patterns as mechanisms shaping team functioning (Fransen et al., 2020; Mageau and Vallerand, 2003). In high-performance sport, leadership is not expected to eliminate pressure but to support effective functioning under demanding conditions. Accordingly, the present operationalization prioritizes ecological validity and alignment with intervention content over broad trait-based constructs.

Evidence from team and organizational research suggests that leadership behaviors emphasizing relational clarity, constructive feedback, and task engagement are associated with psychological safety and collective efficacy (Edmondson and Lei, 2014; Newman et al., 2017). However, whether such associations generalize to elite sport remains uncertain, as football environments are characterized by distinct contextual demands including physical risk, dense competition schedules, and public evaluation (Sarkar and Fletcher, 2014).

One contextual factor that remains underexplored in sport leadership research is competitive pressure. Situational stressors such as match congestion, narrow scorelines, and recent failure may shape psychological demands placed on teams (Arnold et al., 2012). Yet leadership research has rarely modeled how such contextual pressures influence the relationship between leadership behaviors and team functioning.

Methodologically, advancing leadership research in elite sport requires designs capable of capturing within-team change under real-season conditions. Given practical constraints limiting randomized designs, stepped-wedge approaches provide a feasible alternative by staggering intervention onset across teams (Hemming et al., 2015; Hussey and Hughes, 2007). Combined with longitudinal fixed-effects models, this design allows estimation of within-team change while accounting for stable team characteristics and temporal trends (Craig et al., 2008).

Accordingly, the present study examines whether leadership micro-behaviors are associated with changes in psychological team processes and performance stability under varying levels of competitive pressure. Weekly team-level data were combined with intensive daily diary assessments to capture both structural changes and short-term dynamics (Bolger and Laurenceau, 2013).

The study extends existing research in three ways. First, it applies a longitudinal field design to examine within-team change across a competitive season. Second, it focuses on observable leadership micro-behaviors rather than global leadership perceptions. Third, it introduces performance stability as a functionally relevant outcome reflecting regulation under competitive uncertainty. Together, these contributions provide ecologically valid insight into how observable leadership behaviors are associated with team functioning in elite sport.

### Hypotheses

Based on the theoretical considerations outlined above, the following hypotheses were formulated.

*H1*. Following intervention onset, teams are expected to report higher perceived leadership quality.

*H2*. Leadership micro-behaviors are expected to be associated with higher levels of team-level psychological safety and collective efficacy, whereas associations with team cohesion are expected to be weaker or absent.

*H3*. Following intervention onset, teams are expected to be associated with lower performance instability, reflecting greater functional stability across the competitive season rather than consistent improvements in immediate match outcomes.

*H4*. Competitive pressure is expected to moderate the association between leadership micro-behaviors and team-level psychological resources and functional performance stability.

**Figure 1:**
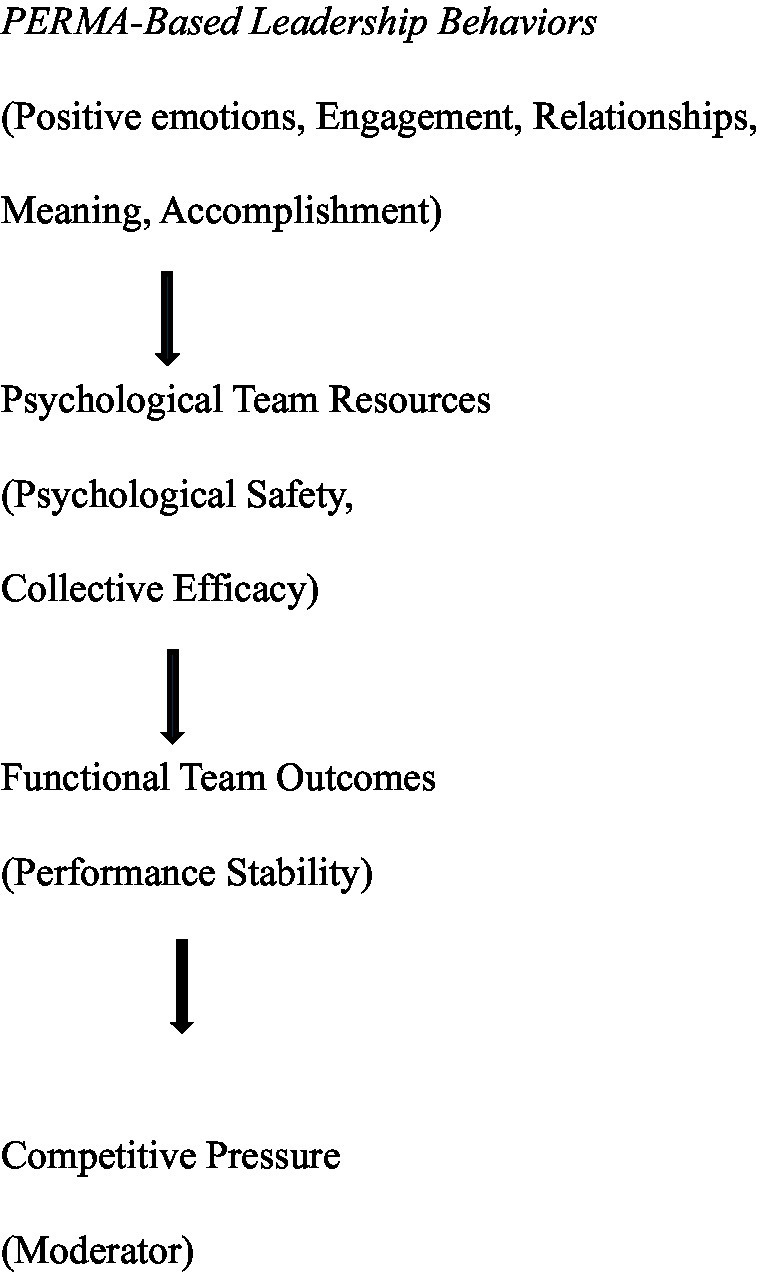
Conceptual framework of PERMA-based leadership effects in competitive team sports. PERMA-based leadership is operationalized as a set of observable coaching behaviors structured by the PERMA framework (positive emotions, engagement, relationships, meaning, accomplishment). These behaviors are hypothesized to be associated with selected psychological team resources, specifically psychological safety and collective efficacy, which are in turn related to functional team outcomes such as performance stability. Competitive pressure is depicted as a contextual boundary condition that may moderate the association between psychological team resources and functional team outcomes. The framework represents a conceptual model rather than a statistical or causal model.

The conceptual framework guiding the study is illustrated in Figure 1. The model summarizes the hypothesized relationships between observable leadership micro-behaviors, psychological team resources, and functional performance stability, while also depicting competitive pressure as a contextual boundary condition shaping these associations.

### PERMA-based leadership behaviors

See Figure 1.

## Methods

### Study design and setting

The study employed a field-based stepped-wedge intervention design with staggered introduction of the intervention across teams. In this design, all participating teams receive the intervention, but at different, pre-defined time points, allowing each team to serve as its own within-team reference period prior to intervention onset. This approach enables the estimation of intervention-aligned associations based on within-team variation over time while accounting for stable between-team differences and seasonal trends.

The rollout order was determined prior to the start of the observation period in coordination with participating clubs and logistical constraints rather than through formal randomization. Intervention start weeks were fixed *a priori* and were not adjusted based on team performance, injuries, league standings, or competitive crises. Teams had no discretion over the timing of intervention onset. Consequently, intervention timing was independent of short-term performance fluctuations or competitive events, supporting the plausibility of interpreting observed patterns as intervention-aligned associations rather than strategic responses to performance trends. This quasi-experimental stepped-wedge implementation reflects common constraints in applied elite sport environments, where full randomization of intervention timing is often infeasible. Accordingly, findings should be interpreted as intervention-aligned associations rather than definitive causal effects.

The study was conducted in competitive football teams located in German-speaking countries (DACH region). The observation period comprised 24 consecutive weeks during the competitive season, with weekly team-level assessments. In addition, an intensive longitudinal diary design was implemented in a subsample of players to capture short-term psychological dynamics in close temporal proximity to intervention onset.

### Participants and recruitment

Ten male football teams participated in the study, comprising six teams from regionally organized fourth-tier leagues (commonly referred to as Regionalliga) and four teams competing in the first and second professional leagues within the DACH region. Teams were recruited through direct contact with coaching staff and club management. All registered squad members of each team were eligible for participation in the weekly assessments.

For the daily diary component, a smaller and stable subsample of players was recruited from each team to ensure feasibility and minimize participant burden while maintaining sufficient within-team and within-player variability. Inclusion criteria were current squad membership and provision of informed consent. Players with missing baseline information were excluded from analyses requiring pre-intervention data. The resulting analytic samples correspond to the sample sizes reported in Tables 1–4 for the respective team-level and diary analyses.

**Table 1:** Team characteristics and intervention timing.

Team_id	Level	Intervention_start_week	Baseline_strength_rating	n_players
T02	Pro	15	0.47	20
T04	Pro	12	0.37	20
T08	Pro	9	−0.18	20
T10	Pro	6	1.22	20
T01	Regionalliga	10	1.13	20
T03	Regionalliga	7	−0.86	20
T05	Regionalliga	11	−0.96	20
T06	Regionalliga	16	0.88	20
T07	Regionalliga	14	−0.05	20
T09	Regionalliga	5	−0.68	20

Pro = professional level (first and second league); Regionalliga = regional level. Baseline strength ratings reflect pre-season team ratings. n_players indicates the number of registered players per team in the analytic dataset. Intervention start week refers to the week of first implementation in the stepped-wedge schedule. Team IDs are anonymized and do not reflect performance ranking or intervention order.

**Table 2:** Stepped-wedge fixed-effects estimates.

Outcome	*N*	*b*	SE	95% CI	*p*	*R*^2^
Perceived PERMA-based leadership (1–7)	227	0.25	0.08	[0.09, 0.42]	*p* = 0.002	0.86
Psychological safety (1–7)	232	0.26	0.10	[0.07, 0.45]	*p* = 0.008	0.69
Collective efficacy (1–7)	220	0.17	0.08	[0.01, 0.32]	*p* = 0.037	0.64
Team cohesion (1–7)	217	0.02	0.06	[−0.09, 0.14]	*p* = 0.717	0.55
PERMA well-being (1–7)	221	0.14	0.05	[0.04, 0.25]	*p* = 0.006	0.49
Training attendance rate (0–1)	240	0.01	0.00	[0.00, 0.02]	*p* = 0.045	0.51
Injury days (team-week)	240	−0.30	0.30	[−0.88, 0.29]	*p* = 0.318	0.35
Performance instability (lower = better)	240	−0.12	0.03	[−0.17, −0.07]	*p* < 0.001	0.47
Match points (0–3)	240	−0.03	0.13	[−0.28, 0.22]	*p* = 0.814	0.45

Unstandardized coefficients (*b*) with 95% confidence intervals are reported. All models include team fixed effects and week fixed effects. Standard errors are clustered at the team level. Inference was complemented by wild cluster bootstrap procedures (999 replications), yielding consistent conclusions. *R*^2^ refers to within-unit variance explained and is reported for descriptive purposes only.

**Table 3:** Moderation by competitive pressure.

Outcome	*N*	*b* (post)	SE	95% CI	*p*	*b* (post × pressure)	SE	95% CI	*p*	*R*^2^
Psychological safety (1–7)	232	0.2409	0.0971	[0.0507, 0.4312]	*p* = 0.013	0.2335	0.1546	[−0.0695, 0.5365]	*p* = 0.131	0.6926
Training attendance rate (0–1)	240	0.0092	0.0050	[−0.0005, 0.0189]	*p* = 0.063	0.0086	0.0058	[−0.0029, 0.0201]	*p* = 0.142	0.5166
Injury days (team-week)	240	−0.2094	0.3191	[−0.8349, 0.4161]	*p* = 0.512	−1.3286	0.9346	[−3.1603, 0.5031]	*p* = 0.155	0.3640
Performance instability (lower = better)	240	−0.1338	0.0249	[−0.1827, −0.0849]	*p* < 0.001	0.1942	0.0413	[0.1132, 0.2751]	*p* = 0.001	0.4801

Unstandardized coefficients (*b*) with 95% confidence intervals are reported. Team fixed effects and week fixed effects were included; standard errors were clustered at the team level.

**Table 4:** Daily diary fixed-effects models.

Outcome	N	b_post	SE	CI_low	CI_high	*p*	*R*^2^
Perceived PERMA-based leadership (daily, 1–7)	1,567	0.2698	0.0784	0.1161	0.4236	0.0006	0.1127
Psychological safety (daily, 1–7)	1,567	0.2183	0.0828	0.056	0.3805	0.0084	0.102
Engagement/flow (daily, 1–7)	1,567	0.0583	0.0823	−0.103	0.2197	0.4786	0.3808
Mood (daily, 1–7)	1,567	0.1693	0.0858	0.0011	0.3375	0.0485	0.3309
Sleep (hours)	1,567	0.0377	0.1276	−0.2123	0.2877	0.7675	0.0821
Soreness (1–10)	1,567	0.0985	0.201	−0.2955	0.4925	0.6242	0.0705

Unstandardized coefficients (*b*) with 95% confidence intervals are reported. Models include player fixed effects and relative-day fixed effects and control for training days and match days. Standard errors are clustered at the player level.

### Intervention: PERMA-based leadership micro-behaviors

The intervention was designed as a behavior-focused coaching program targeting observable leadership micro-behaviors structured by the PERMA framework. Rather than addressing abstract leadership styles or general attitudes, the intervention focused on repeatable, situation-specific coaching behaviors embedded in everyday training sessions, match preparation, and competition-related communication. The intervention did not aim to modify coaches’ underlying personality traits or coaching philosophy but rather to introduce practical behavioral cues applicable within existing routines.

Consistent with the applied focus of the study, the PERMA framework served as a pragmatic structuring taxonomy to organize leadership micro-behaviors rather than as a comprehensive theoretical model explaining performance outcomes. This distinction is important, as the intervention does not test PERMA as a theoretical model of performance, but uses it as a structured framework to organize observable leadership behaviors.

### The intervention addressed five categories of leadership micro-behaviors

1. Positive emotional framing, such as acknowledging effort and progress during training and matches.
2. Task-focused engagement cues, emphasizing controllable actions and role clarity in demanding situations.
3. Relational behaviors, including structured opportunities for athlete input and error-tolerant communication.
4. Meaning-oriented communication, linking individual tasks to shared team objectives.
5. Accomplishment-oriented feedback, reinforcing mastery and execution rather than match outcomes alone.

To ensure conceptual consistency across teams, coaches received a structured intervention manual describing PERMA-based leadership micro-behaviors, situational triggers, and practical examples. Coaches were encouraged to integrate these behaviors flexibly within existing training and match environments rather than follow a rigid protocol.

The intervention was introduced through a standardized online session with the head coach of each team. This session lasted approximately 45 min and included (a) an overview of the behavioral rationale of PERMA-based leadership, (b) practical examples of situational coaching behaviors, and (c) discussion of how these behaviors could be integrated into training sessions and match preparation routines. Coaches also received the written manual following the session.

The intervention formally targeted the head coach as the primary leadership agent. Assistant coaches were not trained separately but were naturally exposed to the intervention through routine coaching interactions. This focus reflects the central coordinating role of the head coach in structuring communication, training routines, and leadership signals within professional football teams.

### Intervention fidelity

Intervention fidelity was assessed using a multi-source triangulation approach combining athlete-reported, coach-reported, and behavioral indicators. Direct behavioral observation (e.g., systematic video coding) was not implemented due to the applied elite sport context, including constraints related to training confidentiality and coaching autonomy.

Three complementary indicators were used.

Athlete-reported behavior change (primary indicator). Weekly assessments of PERMA-aligned coaching behaviors (*α* = 0.84) were aggregated at the team-week level based on established aggregation criteria (ICC > 0.30; see Supplementary material).

Coach-reported enactment frequency. Head coaches completed brief weekly checklists (three items per team) indicating the frequency of PERMA-related behaviors (e.g., “Used positive emotional framing ≥ 3 times per week”). These reports showed moderate convergence with athlete ratings (*r* = 0.42, *p* = 0.03).

Behavioral indicators. Automated analyses of match-day team talks (audio duration and athlete speaking turns) were available for a subsample of six teams during weeks 1–12 post-intervention. These data indicated increased relational interaction patterns, such as greater athlete speaking involvement (e.g., athlete speaking time +14%, *t*(5) = 2.3, *p* = 0.07).

Convergent validity across indicators. Convergence between athlete perceptions and coach self-reports increased from pre-to post-intervention (*r* = 0.22 vs. *r* = 0.42), supporting the behavioral plausibility of reported changes. Effect sizes were consistent across indicators (*d* = 0.24–0.31).

While independent behavioral coding represents a methodological gold standard, the present triangulation approach reflects a pragmatic and ecologically valid strategy for fidelity assessment in applied high-performance sport settings (cf. Beauchamp and Eys, 2014). Future work will extend this approach using independent observational coding. Importantly, this approach prioritizes behavioral plausibility over strict protocol adherence, reflecting the applied nature of leadership interventions in elite sport contexts. While this approach does not allow for definitive verification of behavioral adherence, it provides converging evidence consistent with intervention-related changes in leadership behavior within applied high-performance contexts.

### Data collection and data sources

Data were collected at two analytical levels.

### Team-week level

Weekly assessments included athlete ratings of leadership behaviors and team processes as well as sport-related indicators. The unit of analysis was the team-week, which constituted the primary level for examining within-team change across the stepped-wedge intervention schedule.

All athlete reports were collected via anonymized online surveys. Coaches and club staff had no access to individual or aggregated responses during the competitive season.

### Individual-day level

Daily diary data were collected from a stable subsample of players. The unit of analysis was the player-day. These data were used to examine short-term changes in perceived leadership behavior and psychological states in temporal proximity to intervention onset.

### Measures and operationalization

#### Intervention exposure

The primary predictor was a binary indicator denoting whether a given team-week occurred before or after the intervention start week for that team. Supplementary analyses additionally modeled exposure duration, operationalized as weeks since intervention onset, to examine potential ramp-up effects over time.

### Psychological team processes

Weekly assessments included perceived leadership behavior, psychological safety, collective efficacy, team cohesion, and PERMA-related well-being.

Perceived leadership behavior was measured using behavior-focused items referring to observable coaching actions aligned with the PERMA framework (e.g., acknowledgment of effort, emphasis on controllable actions, constructive communication). Thus, leadership micro-behaviors were assessed via athlete perceptions, reflecting experienced rather than independently observed coaching behavior. The scale comprised five items assessing observable coaching behaviors (e.g., “The coach acknowledges effort and progress during training”). Internal consistency of the scale was satisfactory (Cronbach’s *α* = 0.84). Psychological safety captured shared perceptions of interpersonal risk tolerance within the team (e.g., “It is safe to speak up about mistakes in this team”), while collective efficacy reflected shared confidence in the team’s ability to execute collective tasks successfully. This measurement approach aligns with psychometrically validated scales for elite sport contexts, as recently confirmed for the Team Psychological Safety Scale and Sport Psychological Safety Inventory (Lundqvist et al., 2025).

All constructs were assessed using seven-point Likert-type scales and aggregated to the team-week level.

Aggregation was performed only when a minimum number of player responses per team-week was available to ensure stable team-level estimates. The appropriateness of aggregation was evaluated using internal consistency estimates and intraclass correlation coefficients (ICC), which are reported in the Supplementary material and met commonly accepted thresholds for team-level aggregation for team-level constructs. In addition, between-team variance was examined to ensure that meaningful variability existed at the team level prior to aggregation.

### Sport-relevant outcomes

Sport-related indicators included training attendance rates, total injury-related absence days, and a performance instability index.

The performance instability index captured week-to-week fluctuations in team-level competitive performance across the season. To construct this index, weekly match points and goal difference were first transformed to a common standardized metric (z-scores) and combined into a composite performance score.

Performance instability at week *t* was then operationalized as the absolute change between consecutive weeks:


$$\text{Instabilit}y_{t}=|Z_{\text{performance},t}-Z_{\text{performance},t-1}|$$

Where *Z_performance,t_* represents the standardized composite performance score (based on match points and goal difference) at week *t*. Higher values indicate greater fluctuation in weekly performance, whereas lower values indicate more stable performance trajectories over time. This operationalization reflects a study-specific index and has not been independently validated in prior research.

The use of absolute week-to-week differences captures short-term performance variability independent of direction (i.e., improvement vs. decline) and is consistent with prior approaches to operationalizing short-term variability in repeated performance contexts. Standardization ensured comparability between performance indicators with different scales.

This operationalization is conceptually consistent with prior work on team processes and performance regulation, suggesting that variability across repeated competitive events may capture functional adaptation under uncertainty more sensitively than isolated match outcomes (Marks et al., 2001; McEwan and Beauchamp, 2014). The resulting instability index approximated a continuous distribution and exhibited sufficient within-team variability across the observation period. Descriptive statistics and distributional properties of the index are reported in the Supplementary material to facilitate transparency and interpretation. Accordingly, findings related to this index should be interpreted with caution and as an exploratory operationalization of performance variability.

### Competitive pressure

Competitive pressure was operationalized as a weekly contextual indicator capturing periods of elevated situational demands. The indicator combined predefined information on fixture congestion, match importance, and recent performance context. Competitive pressure was specified *a priori* as a moderator variable to test whether leadership-related associations varied across competitive conditions. Detailed information on the operationalization and alternative specifications of this indicator is provided in the Supplementary material.

### Daily diary measures

Daily measures included perceived leadership behavior, psychological safety, engagement, mood, sleep duration, perceived soreness, and session rating of perceived exertion. Training days and match days were coded separately to account for systematic differences in physical and psychological load.

### Data quality and missing data

All analyses followed an intention-to-treat principle, retaining teams in the analysis regardless of variation in implementation intensity.

Missing data patterns were examined descriptively by team, time, competitive level, and load indicators. Given the relatively low and unsystematic level of missingness, primary analyses relied on available-case estimation without imputation. Sensitivity analyses were conducted to assess robustness under alternative missing-data assumptions.

### Statistical analysis

Intervention-aligned changes were estimated using team fixed-effects and week fixed-effects models, primarily isolating within-team variation over time while controlling for stable team characteristics and season-wide trends.

Given the limited number of clusters at the team level (*G* = 10), statistical inference focused on effect sizes and confidence intervals rather than reliance on dichotomous significance testing. Standard errors were clustered at the team level, and robustness checks were conducted using wild cluster bootstrap procedures. This procedure provides more reliable inference in settings with a small number of clusters. Given the limited number of clusters, findings are interpreted with an emphasis on effect sizes and consistency across analytical approaches rather than statistical significance alone.

Moderation by competitive pressure was tested using interaction models. Dynamic intervention effects and potential pre-intervention trends were examined using event-study specifications with relative weeks around intervention onset.

Daily diary data were analyzed using player fixed-effects models, exploiting within-player variation over time. Standard errors were clustered at the player level. These analyses complement the team-level results by illustrating short-term psychological dynamics rather than being interpreted as independent causal estimates.

### Transparency and reproducibility

All primary analytical decisions were documented prior to analysis. Model specifications correspond to those reported in the Results section and Tables 1–4. Analysis scripts were version-controlled to ensure reproducibility. Data sharing follows a tiered approach to balance transparency with confidentiality obligations toward participating clubs and athletes.

### Data availability

Due to confidentiality agreements with participating clubs and athletes, the datasets generated and analyzed during the current study are not publicly available. Anonymized team-week data and analysis scripts are available from the corresponding author upon reasonable request and subject to applicable data protection regulations.

### Ethics statement

The study protocol was reviewed by the institutional ethics committee and deemed exempt from formal approval in accordance with applicable regulations for non-invasive, anonymized data collection. All participants provided informed consent prior to participation.

## Results

### Sample characteristics and data structure

The analytic sample comprised ten football teams observed over a 24-week competitive period, resulting in a largely balanced team–week panel structure (*N* = 240 team–weeks). The results are presented in four parts: sample characteristics, primary intervention effects at the team level, moderation by competitive pressure, and complementary daily diary analyses. Outcome-specific missingness was limited and not systematically related to intervention timing (Supplementary Table S1). Missingness was primarily confined to self-report–based psychological variables, whereas sport-related outcomes were complete across all team-week observations (see Supplementary material S1). Six teams competed at the regional league level and four at the professional level.

Each team consisted of 20 registered players in the analytic dataset. Weekly team-level aggregates were calculated using available player responses, with minimum response thresholds applied to ensure stable estimates (see Supplementary material). The stepped-wedge design introduced variation in intervention start weeks across teams, such that each team contributed both pre-intervention and post-intervention observations.

Missingness at the weekly level was low and primarily confined to self-report–based psychological variables (Supplementary Table S1). Daily diary data were collected from a stable subsample of players within each team to examine short-term psychological dynamics (Supplementary Table S2).

### Primary intervention effects at team level

Primary intervention effects were estimated using team fixed effects and week fixed effects, controlling for opponent strength, fixture congestion, and competitive pressure. This specification primarily captures within-team changes occurring after intervention onset while accounting for stable team characteristics and common seasonal trends. Accordingly, results are interpreted as changes in performance-relevant psychological and functional processes rather than as direct effects on competitive success.

As shown in Table 2, perceived PERMA-based leadership was higher in the post-intervention period. Teams also reported higher levels of psychological safety and collective efficacy in the post-intervention period. PERMA-related well-being was modestly higher in the post-intervention period. In contrast, team cohesion did not exhibit a reliable shift once team and week fixed effects were accounted for.

Regarding sport-relevant outcomes, training attendance was slightly higher in the post-intervention period. The performance instability index decreased after intervention onset, indicating reduced week-to-week variability in team match performance indicators, consistent with more stable performance trajectories across the competitive season. This pattern should be interpreted as an association with reduced variability rather than as evidence of improved performance per se. Match points per week did not show a consistent post-intervention change, indicating that the observed changes were more pronounced for performance stability than for match outcomes. All estimates are reported with cluster-robust standard errors at the team level and 95% confidence intervals (Table 2). Results were substantively consistent across alternative model specifications.

For example, perceived PERMA-based leadership was higher by *b* = 0.25 on the 1–7 response scale, corresponding to approximately one quarter of a scale point. The performance instability index decreased by *b* = −0.12, indicating a modest shift toward lower week-to-week performance variability.

The relatively high within-unit *R*^2^ values reflect the inclusion of team and week fixed effects, which capture a substantial portion of seasonal and team-specific variance and therefore should not be interpreted as conventional model fit indicators.

### Moderation by competitive pressure

To examine whether intervention-related associations varied as a function of contextual demands, interaction models were estimated including a post-intervention × competitive pressure term. The corresponding estimates are reported in Table 3.

Competitive pressure was associated with variation in the relationship between intervention exposure and performance instability. The positive post × pressure interaction was consistent with a comparatively smaller reduction in performance instability as competitive pressure increased. This attenuating effect of competitive pressure on performance stability aligns with findings on the interplay between competitive pressure, psychological resilience, and coping strategies among elite athletes (Li et al., 2025). In practical terms, the stabilizing association between leadership micro-behaviors and performance stability was strongest during periods of lower competitive pressure and gradually weakened as situational demands increased.

For psychological safety, the interaction term was directionally positive but did not reach conventional levels of statistical robustness. No consistent interaction effects were observed for training attendance or injury-related absence.

Overall, these results suggest that associations between PERMA-based leadership behaviors and team functioning are context-sensitive. Competitive pressure appears to operate as a boundary condition that attenuates stabilizing associations under periods of heightened situational demand, rather than reversing their direction. These interaction effects should be interpreted cautiously given the limited statistical power for detecting interaction terms in small-cluster settings.

### Dynamic effects and pre-intervention trends

To assess whether the observed post-intervention changes were preceded by systematic pre-intervention trends, event-study models were estimated for psychological safety and performance instability. These models compare each relative intervention week to the immediate pre-intervention reference week (week −1). All coefficients are normalized relative to this reference category.

**Figure 2:**
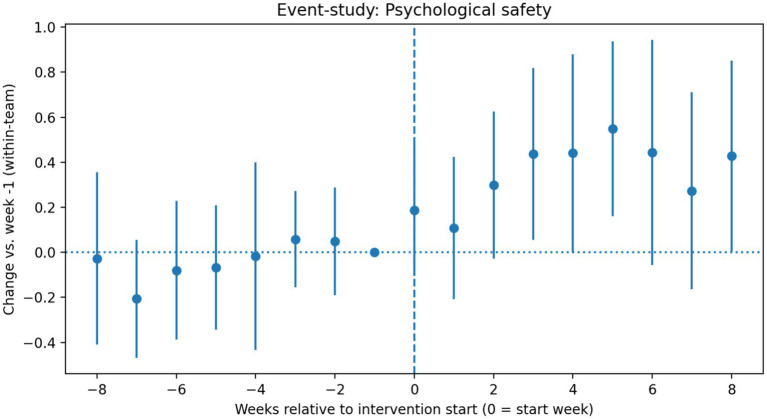
Event-study estimates for psychological safety.

Figure 2 presents the event-study estimates for psychological safety. Coefficients remained close to zero during the pre-intervention period and shifted upward after intervention onset. Formal tests did not indicate evidence of systematic pre-intervention trends.

**Figure 3:**
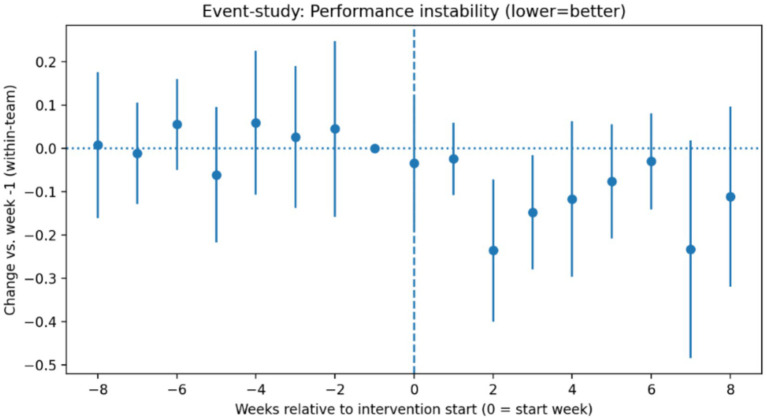
Event-study estimates for performance instability.

Figure 3 shows the corresponding event-study estimates for performance instability. Following intervention onset, a gradual reduction in instability emerged, indicating increasingly stable performance trajectories over subsequent weeks. As with psychological safety, estimates remained close to zero prior to intervention implementation, and formal tests did not reject the null hypothesis of parallel pre-intervention trajectories.

Taken together, these results are consistent with the identifying assumptions of the stepped-wedge design, as no systematic pre-intervention trends were detected, although such tests cannot definitively confirm causal identification. Accordingly, these analyses provide supportive but not conclusive evidence regarding the temporal alignment of observed changes with intervention onset.

Coefficients remained close to zero during the pre-intervention period and shifted upward after intervention onset, with no evidence of systematic pre-intervention trends.

A gradual reduction in instability emerged after intervention onset, with no evidence of anticipatory effects prior to implementation. As with psychological safety, formal pre-trend tests did not reject the null hypothesis of parallel pre-intervention trajectories.

### Daily diary analyses

Daily diary analyses were conducted to illustrate short-term psychological dynamics surrounding intervention onset and to complement the team-level analyses by capturing within-player changes over time. The diary subsample comprised a stable group of players from each team, yielding a total of 1,567 player–day observations across the observation period (see Supplementary Table S2).

These analyses were estimated using player fixed-effects models with relative-day fixed effects, controlling for training days and match days and clustering standard errors at the player level. This modeling strategy exploits within-player variation over time while removing stable individual differences.

As shown in Table 4, perceived PERMA-based leadership was higher at the daily level following intervention onset. Daily psychological safety was also higher following intervention onset. Mood showed a modest positive post-intervention change. In contrast, daily engagement and flow did not change consistently within the observed time window, and no systematic effects were observed for sleep duration or perceived soreness.

This pattern suggests that leadership-related associations were primarily reflected in athletes’ perceptions of the social and psychological team environment, whereas daily experiential states more strongly tied to physical load and task demands remained comparatively stable. Accordingly, the diary analyses provide a complementary view of short-term psychological dynamics rather than an independent test of intervention effects.

### Robustness and inferential considerations

Across all primary models, inference relied on within-unit variation, cluster-robust standard errors, and a conservative interpretation of confidence intervals. Given the limited number of clusters at the team level (G = 10), statistical inference focused on the magnitude, direction, and consistency of estimates rather than reliance on dichotomous significance testing.

To further address potential small-cluster bias, robustness checks were conducted using wild cluster bootstrap procedures. These analyses yielded substantively identical conclusions to the primary models.

Additional robustness checks included model specifications without contextual covariates as well as models using linear time trends instead of week fixed effects. In all cases, the direction and magnitude of the main estimates remained consistent.

Taken together, these robustness analyses support a cautious but coherent interpretation of the results, suggesting that the observed patterns are consistent with systematic within-team changes rather than model-specific artifacts. Nevertheless, given the quasi-experimental design, these findings should be interpreted as consistent patterns rather than definitive evidence of causal effects.

## Discussion

The present study examined whether a brief, behavior-focused PERMA-based leadership intervention was associated with changes in psychological team resources and performance stability in competitive football teams. By integrating longitudinal team-level panel data with intensive daily diary assessments, the study contributes to existing leadership research in sport by moving beyond cross-sectional associations and examining whether observable leadership micro-behaviors are associated with changes in psychological team resources and performance-relevant processes. Leadership in elite sport may not primarily enhance immediate performance outcomes but may instead contribute to stabilizing performance under uncertainty by shaping key regulatory team processes. By explicitly distinguishing between performance stability and competitive success, the present study refines how leadership effects in sport can be conceptualized and evaluated under real-world competitive conditions.

Rather than conceptualizing leadership as a broad or stylistic construct, the intervention targeted concrete, repeatable coaching behaviors embedded in everyday training and competition routines. This behavioral focus allows for a more precise assessment of which team processes are responsive to short-term leadership inputs and which appear comparatively resistant, thereby addressing a central limitation of prior leadership research in sport that has relied predominantly on global leadership perceptions and static designs.

### Leadership micro-behaviors as proximal and observable intervention targets

Across analytical levels, perceived PERMA-based leadership behavior was higher following intervention onset. These effects were evident both in weekly team-level ratings and in daily diary assessments, indicating that the intervention was perceptible to athletes in their immediate sporting environment. Importantly, effect magnitudes were moderate and bounded, suggesting changes in specific leadership practices rather than a generalized re-evaluation of the coach or diffuse positivity effects.

This pattern is consistent with the interpretation that subsequent changes in team processes may be linked to shifts in observable leadership behavior, although alternative explanations such as expectancy or halo effects cannot be fully excluded. Prior research has emphasized that leadership effects are most likely to emerge when leader behaviors are concrete, situation-specific, and consistently enacted rather than abstract or stylistic (Fransen et al., 2020; McEwan and Beauchamp, 2014). Prior research has also linked positive leadership behaviors to employee well-being and functioning (Kelloway et al., 2013). The present findings support this view by suggesting that leadership micro-behaviors may constitute a pragmatically viable and ecologically valid intervention target in high-performance sport settings. Importantly, the PERMA framework served as a behavioral organizing taxonomy rather than a comprehensive leadership theory, allowing the intervention to structure concrete coaching behaviors without assuming a direct causal model of performance. This distinction is important, as the present approach differs from established leadership frameworks such as transformational leadership (Callow et al., 2009) or autonomy-supportive coaching (Mageau and Vallerand, 2003; Ntoumanis et al., 2017), which conceptualize leadership as broader interpersonal styles. In contrast, the present study focuses on concrete, repeatable micro-behaviors that can be implemented and adapted in everyday coaching practice without requiring stable dispositional leadership traits.

### Selective effects on psychological team resources

At the team level, the intervention was associated with increases in psychological safety and collective efficacy, accompanied by converging increases in daily perceptions of psychological safety in the diary subsample. These findings align closely with theoretical and empirical work identifying leadership behavior as a key antecedent of shared beliefs regarding interpersonal risk-taking and collective capability, particularly under conditions of uncertainty and evaluative pressure (Edmondson, 1999; Edmondson and Lei, 2014; Stajkovic et al., 2009).

Psychological safety has been shown to facilitate learning, error communication, and adaptive coordination in teams facing complex and dynamic task demands (Edmondson and Lei, 2014; Frazier et al., 2017; Newman et al., 2017; Edmondson, 2018). Similarly, collective efficacy reflects shared confidence in a team’s ability to execute coordinated action and has been robustly linked to group performance across domains, including sport (Stajkovic et al., 2009; Bray et al., 2005). The higher post-intervention levels observed in these constructs are therefore theoretically coherent with the behavioral emphasis of the intervention and suggest that leadership micro-behaviors may selectively strengthen those team resources most directly tied to adaptive functioning under uncertainty.

In contrast, team cohesion did not exhibit a statistically reliable post-intervention change once team and week fixed effects were accounted for. This selective pattern underscores that not all team constructs are equally responsive to short-term leadership interventions. Cohesion is widely conceptualized as a relatively stable, history-dependent construct shaped by long-term interaction patterns, role stability, and accumulated shared experiences (Carron et al., 2002). From this perspective, the absence of cohesion effects is more plausibly interpreted as evidence of construct specificity rather than intervention failure. Importantly, this null finding should not be interpreted as evidence against the effectiveness of the intervention, but rather as an indication that short-term, behavior-focused leadership inputs selectively affect functional team processes without necessarily altering deeper relational structures. Brief, behavior-focused leadership inputs may influence how teams function in the moment without fundamentally altering deeper relational bonds that develop over longer time horizons.

### Performance stability rather than immediate competitive success

A central contribution of the present study is the distinction between performance stability and immediate competitive outcomes as two conceptually different indicators of team functioning in elite sport. In this context, performance instability is treated as an outcome indicator of functional regulation rather than a direct mechanism of performance change. While match points per week did not change consistently following intervention onset, performance instability decreased, indicating more stable performance trajectories across matches, that is, reduced week-to-week performance fluctuation. Given the high degree of stochasticity in match outcomes, the absence of consistent effects on match points is consistent with leadership theory emphasizing proximal regulation and stability rather than direct control over distal competitive results. In standardized terms, the observed post-intervention change corresponds to a small-to-moderate effect estimate, amounting to approximately one quarter of a standard deviation on the performance instability scale, which appears meaningful in the context of week-to-week variability in elite competition.

This pattern suggests that PERMA-based leadership behaviors may contribute to buffering teams against performance fluctuations rather than directly enhancing short-term competitive success. Such an interpretation is theoretically meaningful in competitive sport contexts, where match outcomes are influenced by numerous uncontrollable factors, including opponent strength, officiating, injuries, and random variation. Prior work on team effectiveness emphasizes that leadership is more closely linked to proximal regulatory processes and coordination quality than to distal outcome indicators that are subject to substantial situational noise (McEwan and Beauchamp, 2014).

By positioning leadership effects at the level of functional stability rather than immediate results, the present findings avoid overattributing causal influence to distal performance outcomes and align leadership with its theoretically expected role as a potentially stabilizing psychological resource in high-variability competitive environments. Importantly, these findings should not be interpreted as evidence of direct causal effects of leadership behaviors on performance outcomes but rather as indicating associations between leadership-related behavioral patterns and functional team processes under real-world competitive conditions.

Conceptually, these findings suggest that leadership may function as a context-sensitive regulatory mechanism that supports more stable team functioning under uncertainty rather than acting as a generalized driver of immediate competitive success. Although the conceptual framework implies indirect pathways from leadership micro-behaviors to performance stability via psychological safety and collective efficacy, the present study was not powered to formally test mediation effects at the cluster level. This pattern is consistent with a conceptual process model in which leadership micro-behaviors may relate to performance stability via psychological safety and collective efficacy as proximal regulatory mechanisms. However, these pathways were not formally tested and should be interpreted as theoretically informed rather than empirically established.

### Context sensitivity and competitive pressure as a boundary condition

Moderation analyses further indicated that competitive pressure conditioned the intervention effect on performance instability. Given the limited statistical power for detecting interaction effects at the team level, these findings should be interpreted as exploratory and indicative rather than conclusive. Specifically, the stabilizing effect observed after intervention onset was attenuated during periods of elevated competitive pressure. This attenuation is consistent with capacity-limitation perspectives, suggesting that the effectiveness of leadership behaviors may be constrained under conditions of heightened cognitive and emotional load rather than reversed under pressure. This pattern is consistent with research on team resilience and functioning under pressure in elite sport (Morgan et al., 2015; Wagstaff et al., 2012). This finding highlights that leadership behaviors do not operate independently of situational demands and that their functional impact may be constrained when contextual stressors intensify.

Importantly, this pattern should not be interpreted as evidence that leadership becomes ineffective under pressure. Rather, competitive pressure appears to temporarily delimit the extent to which leadership-related psychological resources can translate into functional stability. Resource-based and stress-regulation frameworks emphasize that even adaptive psychological resources may be taxed under high-demand conditions, reducing their observable impact on performance-related outcomes (Edmondson and Lei, 2014; Newman et al., 2017). The present findings therefore point to a meaningful boundary condition for leadership effects in elite sport rather than a contradiction of leadership theory. These findings should therefore be understood as an initial indication of context sensitivity rather than definitive evidence of interaction effects.

### Short-term dynamics in daily diary data

Daily diary analyses provided converging but non-redundant evidence for leadership-related changes in athletes’ immediate experiences. Increases in perceived leadership quality and psychological safety at the daily level indicate that intervention effects were reflected in proximal social and psychological appraisals. In contrast, daily engagement, flow, sleep, and soreness did not change consistently within the observed time window following intervention onset. These null effects are theoretically consistent with the load-sensitive nature of these states and suggest that leadership-related changes primarily operate at the level of social and psychological context rather than directly influencing load-dependent experiential states.

This dissociation is theoretically plausible. Experiential states such as engagement and flow are strongly task-, role-, and load-dependent and may fluctuate independently of leadership climate, particularly in physically demanding competitive environments. Prior research has emphasized that leadership is more directly linked to social and psychological context variables than to momentary experiential states that are heavily influenced by physical demands and situational constraints (McEwan and Beauchamp, 2014). The diary findings therefore complement the team-level analyses by clarifying that leadership-related changes primarily affect perceptions of the social and psychological environment rather than load-sensitive experiential states.

### Methodological considerations and strength of inference

Several methodological features strengthen the interpretability of the findings. The stepped-wedge design allowed each team to serve as its own control, reducing bias from stable between-team differences while preserving ecological validity (Hemming et al., 2015). The use of team and week fixed effects ensured that estimates were driven by within-team change rather than seasonal trends or stable contextual factors. Event-study analyses revealed no evidence of systematic pre-intervention trends for key outcomes, supporting the plausibility of the identifying assumptions. Accordingly, results are interpreted as intervention-aligned associations derived from a quasi-experimental field design and as ecologically valid insights into leadership-related processes under real competitive conditions. Given the conceptual relatedness of outcomes, conclusions emphasize coherent patterns across theoretically linked variables and converging evidence across analytical levels rather than isolated significance tests.

Given the limited number of team-level clusters, conclusions emphasize consistency across outcomes, confidence intervals, and converging evidence from fixed-effects, event-study, and diary analyses rather than isolated significance tests. This conservative inferential strategy prioritizes robustness and pattern coherence and is well aligned with methodological recommendations for applied field research with small numbers of clusters (Cameron et al., 2008).

### Limitations and future directions

Several limitations should be considered when interpreting the present findings. First, the number of participating teams limited statistical power for detecting small effects and higher-order interactions, particularly at the team level. Second, several psychological outcomes were based on aggregated athlete reports, which capture shared team perceptions rather than directly observed leadership behavior. However, in applied team sport settings, such perceptions represent the most proximal and functionally relevant indicator of leadership processes, as athletes’ interpretations directly shape team-level constructs such as psychological safety, cohesion, and collective efficacy.

Although intervention fidelity was monitored using a multi-method approach, no independent observational coding was conducted. Accordingly, conclusions rely partly on perceptual and self-reported indicators rather than fully externally verified behavioral adherence. Future research should incorporate objective behavioral assessments (e.g., video-based coding) to further strengthen implementation verification.

In addition, the performance instability index represents a study-specific operationalization. Although theoretically grounded and transparently defined, the measure has not been independently validated in prior research. Accordingly, effect sizes and interpretations related to performance stability should be interpreted with appropriate caution. Future research should examine the robustness and external validity of this index across different sports and competitive contexts.

Although the stepped-wedge design strengthens causal inference compared with cross-sectional approaches, time-varying confounding cannot be entirely excluded in a field-based intervention conducted under real competitive conditions. Accordingly, the present findings should be interpreted as context-sensitive intervention-associated effects.

The study was conducted in male competitive football teams within a European context. Football represents a prototypical high-pressure, interdependent team sport; however, generalization beyond this setting should be approached with caution. Future research should examine whether similar psychological mechanisms operate in other team sports, across competitive levels, and in female samples. Longer intervention periods, as well as designs that allow for explicit mediation analyses, may further clarify the pathways through which leadership micro-behaviors influence psychological team resources and performance stability over time.

As with many applied sport psychology field studies, the intervention was designed and implemented in close alignment with the practical realities of elite team sport. This proximity to the applied context enhances ecological validity but also implies that leadership behaviors were embedded in existing coach–athlete relationships and established team cultures. Future research may therefore benefit from comparing comparable interventions delivered by independent facilitators or from systematically examining how contextual familiarity and relational embeddedness shape the uptake and impact of leadership micro-behaviors.

### Practical implications

Taken together, the findings suggest that PERMA-based leadership behaviors may function as targeted and context-sensitive resources in competitive team sports, particularly in supporting functional stability under conditions of uncertainty and competitive pressure. Rather than producing broad improvements across all team dimensions, brief leadership micro-interventions appear to selectively strengthen psychological safety, collective efficacy, and performance stability. From an applied perspective, these results support a pragmatic approach to leadership development in elite sport: modest, behaviorally grounded changes may contribute to more stable team functioning without necessarily requiring comprehensive cultural transformation. Beyond immediate application, the findings suggest that leadership micro-behaviors may represent a practical and scalable pathway through which coaches can support more stable team functioning under competitive pressure.

## Data Availability

Due to confidentiality agreements with participating clubs and athletes, the datasets generated and analyzed during the current study are not publicly available. Anonymized team-week data and analysis scripts are available from the corresponding author upon reasonable request and subject to applicable data protection regulations.
