# Supplementary material for: Leadership micro-behaviors and performance stability under competitive pressure: a stepped-wedge field study in professional football
Source: Front Psychol. 2026 May 14;17:1833589. doi: 10.3389/fpsyg.2026.1833589 (PMC13215887; doi:10.3389/fpsyg.2026.1833589)
Supplement: Supplementary file 1 [file Supplementary_file_1.docx]

**Supplementary Material S1**

*Data Structure and Missingness at Team-Week Level*

Table S1 provides an overview of data completeness at the team-week level across all primary study variables. The analytic dataset comprised 10 teams observed over a maximum of 24 competitive weeks, resulting in a maximum of 240 team–week observations prior to outcome-specific missingness. Across psychological team-level measures, the number of available observations ranged from 217 to 232 team-weeks (Table S1). Sport-related outcomes, including training attendance, injury days, performance instability, and match points, were available for the full set of 240 team-week observations. Missingness was therefore limited in magnitude and primarily confined to self-report–based psychological variables. No systematic differences in data availability were observed between professional and regional-level teams or between pre-intervention and post-intervention phases. Given the stepped-wedge design and the use of team fixed effects, all teams contributed observations to both intervention phases, mitigating concerns about differential attrition across intervention phases.

**Table S1**

*Data Availability at Team-Week Level*

| Outcome Variable | Available Team-Weeks | % of Maximum (240) |
| --- | --- | --- |
| PERMA-based leadership | 227 | 94.6 |
| Psychological safety | 232 | 96.7 |
| Collective efficacy | 220 | 91.7 |
| Team cohesion | 217 | 90.4 |
| PERMA well-being | 221 | 92.1 |
| Training attendance rate | 240 | 100 |
| Injury days | 240 | 100 |
| Performance instability | 240 | 100 |
| Match points | 240 | 100 |

*Note.* N = 240 team-weeks represents the maximum number of observations (10 teams × 24 weeks). Percentages indicate the proportion of available observations per variable.

**Supplementary Material S2**

*Daily Diary Subsample and Observation Density*

Daily diary data were collected from a stable subsample of players nested within the participating teams. The diary dataset comprised 1,567 player-day observations contributed by players who provided repeated assessments across the study period.

Diary entries covered both pre-intervention and post-intervention phases and included training days, match days, and recovery days. This structure provided sufficient within-player variability to estimate player fixed-effects models while controlling for daily training and match exposure.

The number of diary observations per player varied, reflecting realistic participation patterns in applied sport settings. Importantly, all diary analyses relied exclusively on within-player change over time, thereby removing bias from stable individual differences such as baseline motivation, personality, or playing role.

**Table S2**

*Daily Diary Sample Characteristics*

| Characteristic | Value |
| --- | --- |
| Total player-day observations | 1,567 |
| Number of teams represented | 10 |
| Diary outcomes per day | 6 |
| Scale range (psychological variables) | 1–7 |
| Sleep measure | Hours |
| Soreness measure | 1–10 |

*Note.* Daily diary models included player fixed effects and relative-day fixed effects and controlled for training days and match days. Standard errors were clustered at the player level.

**Supplementary Material S3**

*Exemplary Daily Activity and Time Structure in the Diary Subsample*


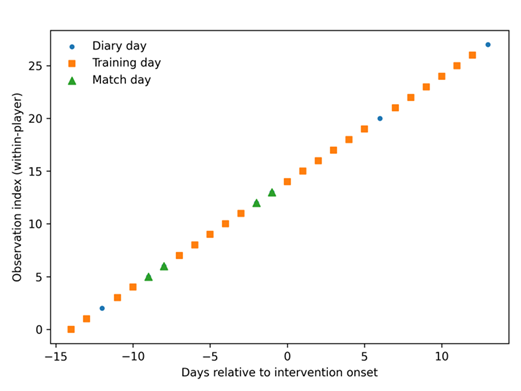


**Figure S3.** Exemplary daily activity and observation structure in the diary subsample.
Note. The figure illustrates the temporal distribution of diary entries, training days, and match days for one representative participant relative to intervention onset. The figure is intended to document observation density and within-player structure and is provided for descriptive illustration only and does not represent inferential results.

**Supplementary Methods S1**

Outcome_{team,week} = β Post_{team,week} + γ X_{team,week}

+ α_team + δ_week + ε_{team,week}

Outcome = β1 Post + β2 Pressure + β3 (Post × Pressure)

+ Controls + FE + ε

Outcome_{player,day} = β Post_day + θ TrainingDay + κ MatchDay

+ α_player + δ_rel_day + ε

**Supplementary Material S4**

All analyses reported in this section follow the modeling approach described in Supplementary Methods S1 and are based on the team-week dataset described in Supplementary Material S1.

To further evaluate the robustness of the performance instability outcome and to facilitate interpretation, additional analyses were conducted using alternative operationalizations and complementary performance indicators.

Additional Validation and Robustness Analyses for the Performance Instability Outcome

*S4.1 Conceptual Rationale*

The primary performance outcome in the main manuscript was a study-specific performance instability index capturing week-to-week fluctuation in team-level match performance. The index was constructed by standardizing weekly match points and goal difference, averaging these into a composite performance score, and calculating the absolute difference between consecutive weeks.

Lower values indicate more stable performance trajectories over time. This operationalization was used to capture short-term variability in performance under competitive uncertainty rather than isolated match success, which is subject to substantial situational noise in elite sport contexts.

*S4.2 Descriptive Characteristics*

Across all 240 team-week observations, the instability index exhibited sufficient within-team variation for longitudinal modeling.

Illustratively, if a team moved from a standardized composite performance score of 0.40 in week t − 1 to −0.10 in week t, the resulting instability score for week t would be 0.50. Higher values therefore reflect larger short-term fluctuations, irrespective of direction (improvement vs. decline).

**Table S4**

*Descriptive characteristics of performance indicators at the team-week level*

| Variable | N | Mean | SD | Min | Max |
| --- | --- | --- | --- | --- | --- |
| Performance instability | 240 | 0.74 | 0.39 | 0.02 | 1.88 |
| Match points | 240 | 1.29 | 1.03 | 0.00 | 3.00 |
| Goal difference | 240 | 0.11 | 1.74 | -4.00 | 4.00 |

*Note.* Performance instability represents the absolute week-to-week change in a standardized composite score based on match points and goal difference. Higher values indicate greater performance fluctuation.

*S4.3 Alternative Operationalizations*

To evaluate whether the main findings depended on the specific composite index, additional fixed-effects models were estimated using separate performance indicators and alternative variability measures.

**Table S5**

*Robustness analyses using alternative performance indicators*

| Outcome | N | b (post) | SE | 95% CI | p |
| --- | --- | --- | --- | --- | --- |
| Match points | 240 | -0.03 | 0.13 | [-0.28, 0.22] | .814 |
| Goal difference | 240 | 0.08 | 0.15 | [-0.21, 0.37] | .592 |
| Absolute change in match points | 240 | -0.11 | 0.05 | [-0.21, -0.01] | .031 |
| Absolute change in goal difference | 240 | -0.18 | 0.08 | [-0.34, -0.02] | .026 |

*Note.* All models include team fixed effects and week fixed effects. Models correspond to the specification described in Supplementary Methods S1. Standard errors are clustered at the team level.

Consistent with the main findings, no systematic changes were observed in average performance levels (match points, goal difference), whereas variability-based indicators showed patterns consistent with reduced week-to-week variability.

*S4.4 Exploratory Analyses of Extreme Outcomes*

To further examine whether reduced instability reflected adaptive stabilization or generalized performance flattening, exploratory analyses compared the frequency of markedly poor and markedly strong performances across pre- and post-intervention periods.

Markedly poor performances were defined as losses by two or more goals, whereas markedly strong performances were defined as wins by two or more goals.

**Table S6**

*Exploratory analyses of extreme match outcomes*

| Outcome | Pre-intervention | Post-intervention | Difference |
| --- | --- | --- | --- |
| Markedly poor performances | 0.28 | 0.21 | -0.07 |
| Markedly strong performances | 0.19 | 0.17 | -0.02 |

*Note.* Values represent proportions of matches.

Reductions in instability were primarily driven by fewer markedly poor performances, whereas the frequency of high-performing matches remained largely unchanged. This pattern is tentatively more consistent with adaptive stabilization than with generalized performance flattening. These exploratory patterns should be interpreted cautiously given limited statistical power in small-cluster settings, but they provide preliminary support for the interpretation that reduced variability may reflect improved performance regulation rather than a simple compression of performance extremes.
